# Dutifully Defying Death: A Right to Life-saving Emergency Treatment

**DOI:** 10.1093/medlaw/fwab009

**Published:** 2021-04-20

**Authors:** Edward Lui

**Affiliations:** BCL Candidate, Faculty of Law, University of Oxford, UK

**Keywords:** International Covenant on Economic, Social and Cultural Rights (ICESCR), International human rights, Life-saving emergency treatment, Resource allocation, Right to health, Socio-economic rights

## Abstract

Article 12 of the International Covenant on Economic, Social and Cultural Rights provides for the right to health. Two questions are considered in this article. Does this right entail a more specific right to life-saving emergency treatment? And if so, should the latter right become justiciable in the domestic courts? Two propositions will be made in this article. First, the right to life-saving emergency treatment is a necessary component of the right to health. Second, the conventional arguments against the justiciability of socio-economic rights do not apply to the right to life-saving emergency treatment. Such a right should be justiciable at the domestic level.

## I. INTRODUCTION

The questions considered by this article can be briefly stated. Article 12 of the International Covenant on Economic, Social and Cultural Rights (ICESCR)^1^ provides for the right to health. It states that ‘[t]he States Parties to the present Covenant recognize the right of everyone to the enjoyment of the highest attainable standard of physical and mental health’.^2^ Does this right entail a more specific right to life-saving emergency treatment? And if so, should the latter right become justiciable in the domestic courts? These questions are clearly of importance: they potentially offer some much-needed protection for deprived patients, and they have yet to be directly confronted in the literature. Two propositions will be made to answer the questions raised. First, there are multiple approaches to formulating the right to health. But whichever approach we adopt, the right to life-saving emergency treatment remains a necessary component of the right to health. Second, we then consider the implications of the first proposition. A conventional view is that a socio-economic right should not be justiciable at a domestic level (ie the right should not be incorporated). This view—insofar as it relates to the right to life-saving emergency treatment—will be refuted. So even assuming socio-economic rights should not be enforceable in domestic law, the right to life-saving emergency treatment should be regarded as an exception: it ought to be enforceable in the domestic courts.

Before we proceed further, two caveats should be noted. First, we are here concerned with the right to health within the ICESCR. However, this does not mean it is the sole potential basis for a right to life-saving emergency treatment. For instance, Nissen concluded that such a right has been recognised in the Strasbourg jurisprudence on the right to life.^3^ This jurisprudence will be touched upon later, insofar as it relates to the right to health. But no attempt will be made to comprehensively evaluate the Strasbourg jurisprudence, as well as Nissen’s claim. Second, this thesis aims to provide a valuable starting point for potentially incorporating a right to life-saving emergency treatment within the UK. It does so by embedding the right within the right to health, and by clearing potential objections against its incorporation. And yet the discussion will *not* be solely limited to the UK context: it applies generally to all dualist states that have ratified the ICESCR. And even within the UK context, clearly much more will have to be done to complete this venture (for instance, which approach towards the right to health should the UK adopt? And should the Human Rights Act 1998 be amended to accommodate this right?). No attempt will be made to answer these questions in this article. These matters are excluded in order to preserve the theme of the current piece: Article 12 of the ICESCR and its potential application *generally* among the states that are subject to it. Incorporating these matters will distract us from this theme: it may lead to the foreseeable objection that the force of this piece is limited to (for the first caveat) states within the Council of Europe and (for the second caveat) the UK. These matters are, in any event, deserving of an article-length treatment and cannot be sufficiently addressed alongside the current discussion.

This article will contribute to the existing literature on medical rationing. Let us take, by way of example, the English literature on this topic. Writing in 2017, Wang noted that the literature has rarely explored the question of medical rationing in a judicial context. This is likely due to ‘the absence of a justiciable right to health written in legislation and a general perception that English courts usually refrain from interfering with discretionary allocative decisions and issues of social policy’.^4^ The development (and the surrounding literature) mostly pertained to an analysis of administrative law, such as rationality and procedural review.^5^ The question of human rights however ‘did not come to the forefront of judges’ legal analysis’^6^—and has indeed been largely neglected by the literature. This article aims to introduce a fresh element into the formula, by paving the way for a justiciable right to life-saving emergency treatment. This article will also contribute to the existing literature of the right to health. Its contribution is self-evident: it argues that the right to health—the specific content of which has been controversial^7^—contains the right to life-saving emergency treatment as an essential component.

## II. THE RIGHT TO HEALTH AND ITS NECESSARY COMPONENT: THE RIGHT TO LIFE-SAVING EMERGENCY TREATMENT

### A. The Right to Health and the Minimum Core

In this section, it will be contended that the right to life-saving emergency treatment is a necessary component of the right to health under Article 12 of the ICESCR. As we shall see, there are multiple approaches towards the right to health in the literature: but whichever approach we take, the states are generally bound to give effect to the right to life-saving emergency treatment. Let us first begin with the minimum core approach. As already mentioned, Article 12 of the ICESCR provides for ‘the right of everyone to the enjoyment of the highest attainable standard of physical and mental health’.^8^ This right translates to the state’s obligation to give effect to the right: and this obligation has a broad scope. It requires not only the states to provide healthcare services (eg healthcare facilities, treatment and drugs): it also requires states to take measures as regards the underlying preconditions for health (eg water, sanitation, food and the environment).^9^ This broad duty must however be read in light of the state’s general obligations under Article 2. It provides that:

Each State Party undertakes to take steps … to the maximum of its available resources, with a view to achieving progressively the full realization of the rights recognized in the present Covenant by all appropriate means.^10^

This means that the state enjoys a significant margin of appreciation in deciding how to give effect to the right to health: and the state’s obligation is subject to resource constraints.^11^ The UN Committee has been quick to clarify that ‘progressive realization’ does not deprive the right to health of any meaningful content. It ‘imposes an obligation to move as expeditiously and effectively as possible towards the goal’^12^—and this obligation is an immediate one.^13^ The worry, nevertheless, is that state compliance cannot be accurately assessed: the states may readily hide behind the veil of resource constraints and claim that it is working towards the right to health, without in fact doing much. This worry has led the UN Committee to take a more radical approach: it introduced the concept of a ‘minimum core obligation’.^14^ The approach means that there is an obligation upon every state to ‘ensure the satisfaction of, at the very least, minimum essential levels of each of the rights’.^15^ Initially, this minimum core obligation will only give rise to a reverse burden: it requires a state to ‘demonstrate that every effort has been made to use all resources that are at its disposition in an effort to satisfy, as a matter of priority, those minimum obligations’.^16^ But later this concept has been developed as an absolute concept: any minimum core obligation must be performed by the state, and this is not subject either to progressive realization and resource constraints.^17^ This approach has not gone without criticism:^18^ and other competing approaches have been proposed. It is beyond the scope of this article to defend the UN Committee’s approach. The thesis here is that, whichever approach we use, the states must generally give effect to the right to life-saving emergency treatment.

### B. The Right to Life-saving Emergency Treatment: A Minimum Core?

If we do apply the UN Committee’s approach, the immediate issue we face is to define the scope of the minimum core obligation under Article 12. This is, admittedly, a difficult task. In *Treatment Action Campaign*,^19^ the Constitutional Court of South Africa was invited to adopt the minimum core obligation approach for the right to have access to health care services under section 27 of the Constitution of the Republic of South Africa. The court refused to do so, citing difficulties to define this minimum core obligation: that ‘in dealing with such matters the courts are not institutionally equipped to make the wide-ranging factual and political enquiries necessary for determining what the minimum-core standards … should be’.^20^ We are not in any better a position. So the objective here will be a less ambitious one: it does not seek to *define* the minimum core obligations exhaustively, it only seeks to ascertain if the right to life-saving emergency treatment forms part of the minimum core obligations.^21^ A prevailing view of the minimum core obligations is to connect them with the basic needs required for survival and life. This is so, because survival is the foundation for every other human right. If one cannot survive, it will be pointless to talk about a right to vote, a right to work, or a freedom of speech. So the attention of the state’s obligations must be, first and foremost, focused on ensuring the survival of its citizens.^22^ This means that the minimum core obligations of the right to health should be focused on the medical needs that are needed for survival, rather than those which are merely facilitative of better health. And the right to life-saving emergency treatment must perforce form part of this core.

Another way to view the minimum core obligations in the literature is to connect them with the concept of human dignity. This view states that human rights are to ensure the respect of human dignity by the state: and correspondingly, the core of such rights will ensure the basic respect for the minimum decencies of citizenship.^23^ Let us expand on this view here. Human dignity is a vague concept:^24^ but its use in the context of socio-economic rights has been developed in the existing literature. Liebenberg’s analysis in this regard is instructive. Human dignity refers to the intrinsic worth of human beings. There is a relational aspect to human dignity: our self-worth is bound up with the extent to which we are valued by others. So when we value the human dignity of human beings, ‘we wish to ensure that conditions are created that enable them to develop their capabilities and to flourish as individuals and social beings’. We may therefore justifiably call upon social resources when we ‘lack the basic material necessities of life to enable [us] to survive and develop as members of the community’. In this regard, the denial of a life-saving medical intervention can ‘indicate a lack of respect for their dignity as human beings entitled to be treated as worthy of respect and concern’.^25^ What follows from this predicates upon a logic similar to the ‘basic needs’ view. If we cannot even survive, we cannot possibly develop as members of the human community: so the protection of human dignity requires survival to first be ensured. It perforce follows that the right to life-saving emergency treatment should be viewed as a minimum core of the right to health, by reason of ‘an imperative of human dignity’.^26^ But it is deceptive to claim that there are two alternative views or lines of analysis. There is in fact only one line of reasoning: that since survival is a logical prerequisite to many other important values—including the exercise of other human rights and the preservation of human dignity—this logical prerequisite must be guaranteed as a minimum core obligation. This is achieved by way of the right to life-saving emergency treatment. Whether the foothold in the reasoning is the existence of other human rights or human dignity does not matter much for current purposes: the thrust of the analysis remains the same.

The foregoing analysis finds affirmation in the General Comment No 14. There the Committee on Economic, Social and Cultural Rights enunciated a list of minimum core obligations that arose from Article 12 of the ICESCR. One can derive indirect support for a right to life-saving emergency treatment from the minimum core obligations in the General Comment. A prominent theme underlying these obligations is the fundamental importance of securing the basic needs for survival: hence the state must ‘ensure access to minimum essential food’ and ensure an ‘adequate supply of safe and potable water’.^27^ If the survival of an individual—secured by the provision of food and water—is important enough to qualify as a minimum core obligation, it seems reasonable for the survival of an individual—secured by the provision of life-saving emergency treatment—to similarly qualify. The foregoing argument finds further support in state practice—which has regarded the right to life-saving emergency treatment as an important facet of the right to health. For instance, the provision of immediate emergency treatments has received repeated mentions in the UK’s periodic reports to the UN Committee on Economic, Social and Cultural Rights. The third periodic report in 1996 emphasised that the provision of access to health care would include efforts to ensure that ‘everyone is able to have immediate accident and emergency treatment, free of charge and within a few miles of his home or of an accident’.^28^ The fourth periodic report in 2001 similarly stressed the need to ensure ‘access to accident and emergency treatment…free of charge to everyone, including visitors to the United Kingdom, regardless of residence status or nationality’.^29^

It may be objected that this thesis proves too much, particularly in the context of the right to health. Bilchitz suggested that imposing a minimum core obligation to ensure survival would mandate the provision of many expensive drugs and treatments which are necessary to preserve life, within each state’s minimum core obligations. This would ‘lead the entire budget of a country to be absorbed by health-care expenditure’.^30^ Tobin has similarly objected that such an approach towards minimum core obligations creates an onerous and impractical burden on the states: and a weaker approach is properly called for.^31^ Both Tobin and Bilchitz appear to assume that many states—particularly those with a small national budget—will be unable to afford the medical expenses involved in a right to life-saving emergency treatments. So such a right will impose an unbearable burden. The conception of ‘resources’ under the ICESCR is however not limited to financial resources. It includes those resources which are available from the international community through international cooperation and assistance,^32^ as well as non-financial resources that are domestically available.^33^ It cannot simply be assumed that many states—once we consider all these available resources—will be unable to answer the burden imposed by regarding the right to life-saving emergency treatment as a minimum core.

There can be two further—and more fundamental—responses to the critic’s objection. First, we can adjust the *consequence* of a minimum core, rather than the definition of the minimum core itself. The current conception of minimum core obligations is absolute and admits of no exceptions. This is to be contrasted with the previous formulation under General Comment No 3, the states are only required to use ‘every effort’ to satisfy the minimum core obligations ‘as a matter of priority’.^34^ So if the states show that they have a genuine grievance in meeting these obligations, they will not be in breach. If we adopt this approach, Bilchitz’s criticism is not inconsistent with prioritizing the meeting of basic needs. The approach will be saying that the states must pour in all their limited resources in meeting the most urgent needs, even if (as Bilchitz suggests) they are expensive. And if the states do not meet these needs, despite all their efforts, they may be excused.^35^ This formulation precisely takes into account the practical limits for any particular state: and so addresses Tobin’s concern.

This analysis finds partial support in the General Comment No 14. It is true that the Comment imposes an absolute form of minimum core obligations.^36^ But other parts of the Comment are consistent with the current analysis. In determining whether there has been a violation of the right of health, ‘it is important to distinguish the inability from the unwillingness of a State party to comply with its obligations under article 12’. So we should separate states which are *unable* to satisfy the right to life-saving emergency treatment from those which are *unwilling* to. Only the latter will be in breach.^37^ Thus, it has been said that the state is only required to ‘take all necessary steps to ensure the realization of the right to health’. While ‘insufficient expenditure … which results in the non-enjoyment of the right to health by individuals or groups, particularly the vulnerable or maginalized’ could amount to a breach of Article 12^38^—and this evidently includes a state’s failure to provide for life-saving emergency treatments—this requirement is not absolute. A mere inability to fulfil the right does not result in a breach. This supports the proposition made in the response: a state can be exonerated, so long as it is genuinely unable to realise the right to life-saving emergency treatment—as part of its minimum core obligations. The Maastricht Guidelines provide even stronger support.^39^ While minimum core obligations ‘apply irrespective of the availability of resources of the country concerned’, it only renders a state *prima facie* in breach of its international obligations.^40^ A state is only in breach through acts of omission if it fails to ‘take necessary measures stemming from legal obligations’, including a ‘failure to utilize the maximum of available resources towards the full realization of the Covenant’.^41^ This posits that if a state has deployed all its available resources—but has still been unable to satisfy the right to life-saving emergency treatment—it will be exonerated. The same is true, even if the right to life-saving emergency treatment is categorised as a minimum core obligation.

Second, even if we do not adjust the consequence of a minimum core, we may say that the right to health *should* impose such an onerous burden over the state budget. When we say that there are resource constraints over the provision of healthcare, there are two possible scenarios. The first is when the state has faced a genuine lack of resources. The second is where the state has had enough resources, but has chosen to invest it elsewhere.^42^ The two matters are not easy to distinguish, but it is clear that the two had a difference. For instance, Perehudoff and Forman have observed that some countries had a staggering level of military spending when compared that to health—even by the ratios of 20:1 and 38:1.^43^ While military spending is to an extent necessary, it is at least contestable that the state *should* be required to redirect such resources—at least in part—to maintain the basic survival of its citizens. This coincides with the normative basis of the socio-economic rights: to introduce a just distribution of resources and to provide for a minimum floor of rights, particularly for the poor and the oppressed.^44^ And this is an ideology which a state would have subscribed to, when it signed and ratified the ICESCR. Viewed in this light, the fact that the minimum core approach would impose an onerous burden over the state budget is commendable, rather than objectionable.

### C. A Reasonableness Approach Towards the Right to Health

As mentioned above, the minimum core approach has not been universally welcomed. For instance, South African law has refused to follow the minimum core approach. Instead, it has adopted a general standard of reasonableness.^45^ It may be objected that even if the right to life-saving emergency treatment constitutes a minimum core, this is contingent upon a contestable premise: that we should adopt the minimum core approach within the ICESCR. In other words, the critic may argue that once we reject the minimum core approach within the ICESCR, the foregoing argument will fall away. This, however, does not suffice to reject the right. For even if we reject the minimum core approach, we must still adopt *an* approach for applying the right to health under Article 12. One such notable candidate is the reasonableness approach, which was itself adopted in South Africa as an alternative to the minimum core approach.^46^ As we shall see, while the concept of reasonableness does not readily produce an *absolute* position—ie a matter is *always* required by the concept—it does not mean there is no certainty in it. Scholars have distilled several guiding principles that the South African courts can act upon in applying the concept of reasonableness.^47^ It will be contended that—properly analysed—the reasonableness approach leads us to a general right to life-saving emergency treatment.

It is noteworthy that the reasonableness approach does not neglect the basic needs of the people. Pillay has analysed the South African jurisprudence, and found that the reasonableness approach takes into account four principal factors. They are the constitutional balance of power, relative institutional expertise, the severity of the impact of the government action or inaction, as well as state conduct.^48^ The first two factors mean that the court will take into account resource constraints. The allocation of resources is, according to this view, matters of socio-economic policy which should be primarily decided by the legislature and executive—whether as a matter of constitutional legitimacy, or as a matter of institutional expertise.^49^ Almost every case concerning the right to health will involve a defence by the Government along the lines of resource constraints. So these two factors are almost always part of the reasonableness assessment in every right to health case. What distinguishes one case from the other is not the existence of these factors, but the weight attached to them. For instance, if the cost implications of the measure are known and relatively moderate, these factors will play a lesser role.^50^

When we come to the third factor, the courts will examine how the applicant is being affected.^51^ A serious impact upon the applicant’s dignity,^52^ or upon their prospects of survival,^53^ will justify a less deferential approach. This means that the reasonableness approach is—just as the minimum core approach—sensitive to the basic needs and human dignity of the affected patients. And these matters are, as explained above, precisely what underlies the recognition of a right to life-saving emergency treatment. Thus Forman has suggested that:

[T]he reasonableness standard is not theoretically inconsistent with the minimum core. While the standard is amorphous, it nevertheless gives important guidance on health policy, by requiring comprehensive policies focused on basic needs of the poor and vulnerable, and on urgent and desperate needs.^54^

This focus requires, therefore, that the right to life-saving emergency treatment be accorded priority in the state’s healthcare policy. When there is a failure to provide such treatments, there is a gross violation of the patient’s basic needs and dignity: and this must attract great weight towards the third factor in the particular case. It is fairly accepted that this is not an *absolute* position. Even if a life-saving emergency treatment is denied, the measure may be overridden by the first and second factors—if the cost implications involved in the provision of such treatments are sufficiently immense or uncertain. This is so in the famous case of *Soobramoney*.^55^ There the applicant—who suffered a renal failure—was refused renal dialysis. The court knew full well the grave impact that the refusal would cause to the applicant. Nevertheless, it ruled that the refusal was reasonable. This, as Pillay analysed, was crucially because the cost implications of allowing the applicant (and the persons in his position) renal dialysis were unclear—and this uncertainty overrode the third factor.^56^

But the measure *will* fall foul of the reasonableness approach, unless there is a sufficiently weighty concern under the first and second factors. This is illustrated by two cases. In *Treatment Action Campaign*, the Government deliberately limited access of a drug that can prevent the mother-to-child transmission of HIV by the general public. The court ruled that the policy was unreasonable. The third factor was engaged on the facts—since the policy denied a potentially life-saving drug to the general public, particularly mothers and children who did not have access to private health care. Importantly, the resource constraint factors did not override the third factor here: for the cost of distributing the drug to the overall population was minimal—and indeed well within the State’s available resources.^57^ In *Khosa*,^58^ some destitute permanent residents challenged the social security system which excluded them. The Government opposed the challenge, by reason of its cost implications. The court held that this exclusion would inevitably force these permanent residents into dependence on their families. This, said the court, was ‘likely to have a serious impact on the dignity of the permanent residents’^59^ and thus lead to ‘grave’ consequences.^60^ This engaged the third factor. Importantly, the court noted that including such members within the social security system would only lead to an increase of less than 2% in social security spending. This, remarked the court, was ‘only a small proportion of the total cost’.^61^ With only clear and moderate costs on the first and second factors, the court found the exclusion unreasonable.

It is submitted that the above mentioned cases are instructive. They illustrate how the factors interacted with each other when there is a gross violation of basic needs or human dignity—of which life-saving emergency treatment is an example—and such an interaction may be expressed as follows. There is, *prima facie*, a failure to meet the reasonableness approach whenever a life-saving emergency treatment is denied. And this *prima facie* position may be displaced by a sufficiently weighty concern over resource constraints. This, in effect, recognises a *general* right to life-saving emergency treatment—even if we apply a reasonableness approach. It is true that this general right is different from an *absolute* right under the minimum core approach. But this analysis shows, at least, the critic cannot succeed by simply eschewing the concept of minimum core obligations. The right to health—however formulated—provides a foundation for the right to life-saving emergency treatment.

It may be objected that subjecting the right to life-saving emergency treatment to a reasonableness approach will denigrate its very status as a right. Under this formulation, so the objection goes, the provision of life-saving emergency treatment can be readily overridden by mere resource concerns. This offers so much leeway to the state that it renders the provision of life-saving emergency treatment a mere, admirable policy aim—as opposed to a human right. This is not necessarily true, for three reasons. First, the mere fact that X can be overridden by resource concerns does not imply that X is not a right. For rights—even civil and political ones—can be justifiably qualified by resource concerns under the proportionality test.^62^ It does not follow that they are no longer rights.

Second, the reasonableness approach—as formulated above—does more than merely setting out a policy aim. Like a policy aim, the approach towards the right to life-saving emergency treatment sets out a default position that a state ought to follow. But unlike a mere policy aim, this default position cannot be easily departed from. Cases such as *Treatment Action Campaign* and *Khosa* show that reasonableness is not a subjective, arbitrary standard that the state can satisfy whenever the words ‘resource allocation’ are invoked. What this entails is that the state must—in order to satisfy the reasonableness standard—objectively *justify* its failure to provide for life-saving emergency treatments. This burden to justify is by no means negligible: indeed, Fredman has explained that ‘[u]nder the reasonableness review, the Court will not inquire whether there are other more desirable measures which could have been adopted. *However, reasonableness demands a high level of scrutiny of the State’s justifications*’.^63^ This paints a very different picture from a mere policy aim that can be departed from with relative ease.

Third, even if one disagrees with Fredman’s depiction of the reasonableness approach, a low degree of scrutiny is not necessarily inconsistent with the existence of a right. Because a right does not always entail hard limits and strenuous controls on the state. Rights-enforcement can play a supplementary role to the executive and the legislature: it can accept that there can be room for reasonable disagreement in applying the right, while insisting on reasons to be given by the decision-maker (this point will be developed further below).^64^ This is consistent with the current formulation of the right to life-saving emergency treatment: even if the reasonableness approach does not impose a rigorous standard, a state will at least be required to justify its stance before a court. This is, again, different from a mere policy aim—which generally does not impose a duty to give reasons on the decision-maker.^65^

## III. THE RIGHT TO LIFE-SAVING EMERGENCY TREATMENT: THE CASE AGAINST JUSTICIABILITY

So the right to life-saving emergency treatment is a necessary component of the right to health under Article 12 of the ICESCR. But this right—as an international human right—does not automatically apply to the domestic legal order. For in a dualist state (such as the UK), international treaties are not justiciable before domestic courts—in the absence of any incorporation through domestic legislation.^66^ An objection may then be as follows: even if we accept that the right to health contains a right to life-saving emergency treatment, the incorporation of the former (including the latter as its sub-set) should be resisted. For these rights are socio-economic rights: and such rights, normatively speaking, should not be justiciable in domestic courts. To this end, the critic may invoke the conventional arguments against the justiciability of socio-economic rights. So any attempt to incorporate a right to life-saving emergency treatment should be resisted. In other words, the critic accepts the premise that the right to life-saving emergency treatment exists within the right to health at the ICESCR level: but she contests the implications of this premise. This objection does not only relate to UK law: it may, if valid, apply to all dualist states.^67^ It will be contended that this objection is wrong. Even assuming that the conventional arguments against the justiciability of socio-economic rights are generally valid, they do not preclude the justiciability of a right to life-saving emergency treatment in domestic courts.^68^ (But no position will be taken in respect of the justiciability of the right to health as a whole here). Two such conventional arguments will be addressed. Like the objection itself, the responses below do not exclusively relate to UK law: they may apply to dualist states generally.

First, a conventional argument against the justiciability of social and economic rights is that their scope is vague. They are also not framed a way that may be capable of immediate enforcement.^69^ For instance, Article 15 of the ICESCR recognises the right of everyone to ‘enjoy the benefits of scientific progress and its applications’.^70^ Article 12 can also be regarded as vague: one may understandably question what the ‘highest attainable standard of physical and mental health’ means. As Maxwell pointed out, this language ‘often resembles policy documents or a political manifesto’—and poses a particular set of problems.^71^ One may note at the outset the general weakness of this argument: it is outdated. The argument is outdated, because the more recent developments of socio-economic rights have gradually mitigated the problem of vagueness. For instance, the General Comments (such as the ones referred to above) provide much clarification of the content of the state’s obligations under the ICESCR. The introduction of the Optional Protocol to the ICESCR also allows for potential claims of violations to be decided upon.^72^ Other bodies can also shed light on the content of socio-economic rights. One example is the European Committee of Social Rights. It may clarify the content of socio-economic rights under the European Social Charter^73^ through its case law.^74^ It may also adopt interpretations of socio-economic rights under the Charter. The jurisprudence of the Committee can potentially provide valuable guidance for rights under the ICESCR. For instance, in its recent interpretation on the right to health under the European Social Charter, the Committee issued valuable guidance of what the right entails in a pandemic. It requires states to take all necessary emergency measures in a pandemic (eg through testing, tracing and enforcing isolation), to take all necessary measures to treat those who fall ill in a pandemic (eg by ensuring the availability of hospital beds, intensive care units and equipment), and to take precautionary measures against the pandemic.^75^ There is no reason why such guidance should be regarded as irrelevant for the right to health under the ICESCR.

But regardless of whether the conventional argument applies to social and economic rights generally, it is not sustainable against the right to life-saving emergency treatment. As Jheelan argued, the vagueness of a right is not an excuse for non-justiciability. The proper response is instead to define the right through precedents.^76^ For instance, much certainty has been instilled into the content of the right to life-saving emergency treatment in *Lopes* (assuming Nissen was right to say that such a right existed in the Strasbourg jurisprudence on the right to life^77^). There the court concluded that under two ‘very exceptional circumstances’, the state may be found in breach of its positive obligations under Article 2 for a failure to provide to life-saving emergency treatments.^78^ The first category of circumstances is ‘where an individual patient’s life is knowingly put in danger by denial of access to life-saving emergency treatment’.^79^ The second category of circumstances is:

[W]here a systemic or structural dysfunction in hospital services results in a patient being deprived of access to life-saving emergency treatment and the authorities knew about or ought to have known about that risk and failed to undertake the necessary measures to prevent that risk from materializing, thus putting the patients’ lives, including the life of the particular patient concerned, in danger.^80^

These requirements are concrete and detailed. While *Lopes* does not directly relate to the right to health under the ICESCR, it shows us that vagueness is not a necessary concomitant of the right to life-saving emergency treatment. One may nevertheless criticise that there are some remaining problems with the formulation in *Lopes*. For instance, the phrase ‘life-saving emergency treatment’ has not been clearly defined throughout the authorities and the literature.^81^ But this does not detract from the force of the current argument. If one follows Jheelan’s position, one should go even further and define this expression through further adjudication. And in any event, even with this ambiguity in mind, the right to life-saving emergency treatment is significantly much clearer than the content of other civil and political rights. An immediate example that comes to mind is the positive obligations to protect against forced labour under the European Convention on Human Rights and the International Covenant on Civil and Political Rights.^82^ The authorities essentially require the state to provide ‘practical and effective protection’ against forced labour—without giving much specific content to what that expression means.^83^ But no one would say, for this reason, that such a right should not be enforceable in our courts altogether.

Second, another conventional argument against the justiciability of socio-economic rights is based on resource allocation. The argument goes as follows. Unlike that of civil and political rights, the adjudication of socio-economic rights will inevitably require resource allocation. And the courts are neither constitutionally legitimate—nor institutionally competent—to adjudicate on such matters. It is better that these matters be dealt with by the Government and Parliament.^84^ The common response is that courts frequently adjudicate on matters that impact upon resource allocation: and this is most certainly true when civil and political rights are adjudicated upon.^85^ But this proposition proves too much. It ignores the fact that there can be a degree of difference between cases involving civil and political rights, and those involving socio-economic rights. When the former is being enforced, one can easily imagine a case that carries little cost implications. For instance, although the establishment of a criminal justice system itself is expensive, a judicial decision to enforce the right to *habeas corpus* for an individual prisoner generates little cost implications.^86^ But one can easily imagine a case where a court decides to enforce socio-economic rights—and this significantly impacts on the national budget (recall *Soobramoney*). This will, *per* the conventional argument, raise a strong need for judicial deference. One cannot simply say that since the courts adjudicate on (say) the freedom of assembly notwithstanding the (limited) cost implications, the courts should equally adjudicate on (say) the right to health in an identical manner. This confuses the *existence* and *extent* of cost implications. One must not however over-generalise: this is not to say that civil and political rights *always* attract fewer cost implications than socio-economic rights. If the enforcement of civil and political rights has significant cost implications (eg a positive duty to establish the criminal justice system itself), it will similarly attract a need for judicial deference.^87^ The contrary is also true: if the enforcement of socio-economic rights does not attract much cost implications (recall *Treatment Action Campaign*), the argument for judicial deference is significantly weaker. Nevertheless, the force of the conventional argument cannot be entirely dismissed. While one cannot readily demarcate civil and political rights from socio-economic rights (which the argument wrongly suggests), the argument rightly suggests that the adjudication of socio-economic rights can potentially draw upon a need for judicial deference. And whilst this is not inevitably the case when socio-economic rights are enforced, it is certainly not a rare occurrence.

But a distinction must be drawn between *mere* judicial deference and non-justiciability.^88^ Even when this conventional argument justifiably calls for judicial deference, it does *not* follow that socio-economic rights should be non-justiciable altogether. We have seen this from the South African approach (and Pillay’s analysis of it). There the courts took into account the need to defer to resource allocation decisions: but balanced it with the need to protect the applicants against any particularly harsh consequences. Many supporters of the justiciability of socio-economic rights also take this stance. For instance, Fredman has accepted that the ‘[p]rimary responsibility for determining how to fulfil positive duties should lie with the legislature and executive’: or otherwise it will undermine democracy.^89^ Nevertheless:

While judges should not have power to make primary decisions as to how resources should be allocated, they should be able to demand that decision-makers give a deliberative account of the use of ‘available resources’ to meet their human rights obligations.^90^

This is done by adjudicating on socio-economic rights: so that the decision-makers will be required to justify their decisions and convince the courts. This means that the court is only complementing Parliament’s role concerning resource allocation: it does not eclipse it.^91^ This approach respects—and is consistent with—the constitutional imperatives to defer on resource allocation grounds. The right to life-saving emergency treatment is in line with this approach. Let us again recall that this right has been said to exist in the Strasbourg jurisprudence on right to life.^92^ In *Lopes*, the court discussed this right and emphasised that:

[I]ssues such as the allocation of public funds in the area of health care are not a matter on which the Court should take a stand and that it is for the competent authorities of the Contracting States to consider and decide how their limited resources should be allocated, as those authorities are better placed than the Court to evaluate the relevant demands in view of the scarce resources and to take responsibility for the difficult choices which have to be made between worthy needs.^93^

This passage shows two things in the court’s approach. First, the court—like Fredman suggests—does not play a primary role towards the allocation of resources in healthcare. Its role is complementary only. That is why the court was at pains to emphasise that the two categories of the right to life-saving emergency treatment are ‘very exceptional circumstances’.^94^ Second, the court is clearly aware that the relevant authorities are ‘better placed’ to assess matters of resource allocation.^95^ This is in line with the aforementioned need to defer over resource allocation matters, by reason of a deficit in institutional competence and constitutional legitimacy. If these two things are true of a right to life-saving emergency treatment derived from the right to life, they can be equally true of a similar right derived from the right to health. For these reasons, the argument based on resource allocation is respected by—and accordingly does not preclude—a right to life-saving emergency treatment.

## IV. AN ILLUSTRATION: COVID-19 IN THE UK

The core propositions of the thesis have been stated above. This section seeks to provide an illustration of how the right to life-saving emergency treatment may apply in practice. Let us take, by way of example, the UK Government’s obligations in the face of COVID-19. This example will naturally focus on the UK context. But the analysis which follows is not intended to be exclusive to UK law: it relates to an international obligation that applies—among many others—the UK, and may (with the necessary adjustments made) apply to other states under the ICESCR. Say Tom has been infected with COVID-19. Upon initial medical assessment, he was tested positive and was found to be at a very substantial risk of death from COVID-19. But when he arrived at the hospital, no life-saving emergency treatment was available for him. This was because all hospital beds in the NHS nationally had already been occupied, and he must wait until there was a vacancy. During the wait, Tom died from COVID-19. Has the UK Government been in breach of Tom’s right to life-saving emergency treatment under Article 12 of the ICESCR (assuming this right is now justiciable in the English domestic courts)?

Before this question can be answered with more certainty, we must first further specify the approach we are taking towards the right, as well as the factual circumstances underlying the failure. If we apply the minimum core approach (in its earlier form in the General Comment No 3), the UK Government will have *prima facie* breached Tom’s right to life-saving emergency treatment. It will however be availed if it demonstrates that ‘every effort has been made to use all resources that are at its disposition in an effort to satisfy, as a matter of priority, those minimum obligations’.^96^ The Government may argue that it has done so, by showing that it has already exhausted its budget for the NHS. There may be a strong argument that the UK Government is indeed in genuine financial difficulties, given the many financial demands on it (eg the furlough scheme).^97^ One must however bear in mind Skogly’s analysis—the state’s resources are not limited to financial resources. Thus the UK Government must also show, *inter alia*, that it has exhausted its human resources^98^ to create more empty hospital beds—through engaging medical students and retired medical professionals for service in the NHS,^99^ and through engaging its human resources for creating more Nightingale Hospitals (eg the UK Government’s use of military personnel to support the opening of Nightingale Hospitals^100^). In contrast, if we apply the absolute form of the minimum core approach in the General Comment No 14,^101^ the UK Government will have been in breach of Tom’s right to life-saving emergency treatment—quite regardless of its endeavours.

Let us then apply the reasonableness approach to Tom’s scenario. There is a *prima facie* breach of the right to life-saving emergency treatment, whenever such a treatment has been denied. The question is whether this *prima facie* position has been displaced by a sufficiently weighty concern over resource constraints. This could be the case when the cost implications of such treatments are sufficiently immense or uncertain. Unlike the minimum core approach, it is unclear if Skogly’s analysis can apply to the reasonableness approach. Assuming it does not, the UK Government appears to have a strong argument: it has been reported that the NHS lacks over £1 billion to deal with the second wave of COVID-19.^102^ Or at least, the Government may argue that the cost implications of allowing Tom (and persons in his position) life-saving emergency were uncertain, by analogy to *Soobramoney*.^103^ The analysis in this section illustrates the usefulness of having a right to life-saving emergency treatment. The absolute form of the minimum core approach will evidently protect applicants that have had their basic needs and human dignity denied. Yet as we have seen, this right in other forms does not always avail an applicant who has been denied life-saving emergency treatment. Such a right will nevertheless be useful. For it will consistently require the Government to justify its failure to provide such treatments, through providing evidence of how it has sought to meet its obligations and other demands of its limited resources. If the Government fails to provide a satisfactory justification, the court can nevertheless find the right to be breached.

## V. CONCLUSION

In conclusion, two propositions have been made. First, the right to life-saving emergency treatment is a necessary component of the right to health under Article 12 of the ICESCR. Second, the conventional arguments against the justiciability of socio-economic rights do not apply to the right to life-saving emergency treatment. It is important to clarify what follows from these two propositions. This article accepts that the right to health exists as an international human right: and is, without any incorporation, non-justiciable in domestic courts. Thus, at present, the right to health (or any component of it) is *not* justiciable within the UK courts. Against this background, this article argues that the right to life-saving emergency treatment exists within the state’s international obligations under the right to health in the ICESCR. It further argues that such a right should be incorporated into the state’s domestic law—and become justiciable in the courts. However, as made clear by the caveats earlier, this piece does not exclusively relate to the UK. The ‘state’ can be the UK, but it can be other states that have ratified that ICESCR and are dualist in nature. This piece however does not seek to resolve whether the UK should opt for either the minimum core approach or the reasonableness approach, when there is such an incorporation. What is now suggested is simply that either approach to the right to health leads us to the right to life-saving emergency treatment.

It has been mentioned earlier that the right to life may contain a right to life-saving emergency treatment as well.^104^ This article is evidently not a detailed or comprehensive exposition of the right to life: but two further points about it that concern the right to health are noteworthy. First, the use of the right to health may potentially achieve something we could not have achieved with the right to life. The right to life is limited to treatments that maintain the survival of a person. It cannot go further to provide for a right to treatment aside from those necessary for survival.^105^ This is something that the right to health, if further developed, can achieve. In this sense, basing the right to life-saving emergency treatment on the right to health can be seen as a first step towards a developing a wider right to treatment. Second, the foregoing analysis shows that a divide of justiciability based on whether a right is civil and political or socio-economic is hardly tenable. If Nissen is correct,^106^ both the right to life and the right to health contain a right to life-saving emergency treatment. Even if the content of this specific right is not identical on both occasions, there will inevitably be an overlapping content. If one regards a matter as properly justiciable under the label of the right to life, it should equally be justiciable under the label of the right to health. This conclusion can only be achieved, if one abandons a strict instance on the divide between civil and political rights and socio-economic rights. This corroborates Fredman’s analysis: we should not focus on whether a right is labelled as civil and political orsocio-economic; what is important is to understand the nature of the right’s positive obligations.^107^

Acknowledgements

I would like to thank Josh Baker, Tom Chan, Professor Jonathan Herring, Thomas Yeon and the anonymous reviewers.

Conflict of interest statement: I have no conflict of interest to declare.

## Footnotes

1 International Covenant on Economic, Social and Cultural Rights (New York, 16 December 1966, entered into force 3 January 1976, 993 UNTS 3).

2 ibid art 12(1).

3 A Nissen, ‘A Right to Access to Emergency Health Care: The European Court of Human Rights Pushes the Envelope’ (2017) 26(4) Med L Rev 693, 697.

4 DWL Wang, ‘From Wednesbury Unreasonableness to Accountability for Reasonableness’ [2017] Cambridge L J 642, 642–43.

5 C Newdick, *Who Should We Treat? Rights, Rationing and Resources in the NHS* (2nd edn, OUP 2005) 100–7; ibid 648–52; K Syrett, ‘Healthcare Resource Allocation in the English Courts: a Systems Theory Perspective’ (2019) 70 Northern Ireland Legal Quart 111, 114–16.

6 Wang (n 4) 656.

7 See, eg K Hessler and A Buchanan, ‘Specifying the Content of the Human Right to Health Care’ in A Buchanan (ed), *Justice and Health Care: Selected Essays* (OUP 2009); J Tobin, *The Right to Health in International Law* (OUP 2012) Chs 6–7.

8 ICESCR (n 1) art 12(1).

9 R Sifris, ‘Restrictive Regulation of Abortion and the Right to Health’ (2010) 18 Medical L Rev 185, 192; Tobin (n 7) 50.

10 ICESCR (n 1) art 2(1).

11 Tobin (n 7) 124, 179, 197–8.

12 Committee on Economic, Social and Cultural Rights, CESCR General Comment No 3: The Nature of States Parties’ Obligations (art 2, para 1, of the Covenant) (14 December 1990) (‘General Comment No 3’) [9].

13 S Fredman, ‘New Horizons: Incorporating Socio-economic Rights in a British Bill of Rights’ [2010] Public L 297, 314, 316.

14 Tobin (n 7) 242; P Thielbörger, ‘The “Essence” of International Human Rights’ (2019) 20 German L J 924, 934.

15 General Comment No 3 (n 12) [10].

16 ibid.

17 Committee on Economic, Social and Cultural Rights, CESCR General Comment No 14: The Right to the Highest Attainable Standard of Health (art 12) (11 August 2000) (‘General Comment No 14’) [47]; *Lopes de Sousa Fernandes v Portugal* (2018) 66 EHRR 28 [OI-6], [OI-28].

18 Tobin (n 7) 243–47.

19 *Minister of Health v Treatment Action Campaign* 2002 (5) SA 721 (CC).

20 ibid [37]. See also *Government of the Republic of South Africa v Grootboom* (2001) (1) SA 46 (CC) [32]; *Mazibuko v City of Johannesburg* (2010) (4) SA 1 (CC) [56]–[68].

21 The relationship of the right to life-saving emergency treatment with the South African approach—which rejected the minimum core approach—will be dealt with later.

22 KG Young, ‘The Minimum Core of Economic and Social Rights: A Concept in Search of Content’ (2008) 33 Yale J Int L 113, 128–30; L Forman, ‘What Future for the Minimum Core? Contextualising the Implications of South African Socioeconomic Rights Jurisprudence for the International Human Right to Health’ in J Harrington and M Stattaford (eds), *Global Health and Human Rights: Legal and Philosophical Perspectives* (Routledge 2010) 68.

23 Young (n 22) 135; Forman (n 22) 69; *Lopes* (n 17) [OI-24].

24 R Macklin, ‘Dignity Is a Useless concept’ (2003) BMJ 1419.

25 S Liebenberg, ‘The Value of Human Dignity in Interpreting Socio-Economic Rights’ (2005) 21 South African J Human Rights 1, 11–13.

26 *Lopes* (n 17) [OI-71].

27 ibid [43]–[44].

28 United Kingdom of Great Britain and Northern Ireland, *Implementation of the International Covenant on Economic, Social and Cultural Rights: Third periodical reports submitted by States parties under articles 16 and 17 of the Covenant (Addendum)* (17 June 1996) [210].

29 United Kingdom of Great Britain and Northern Ireland, *Implementation of the International Covenant on Economic, Social and Cultural Rights: Fourth periodical reports submitted by States parties under articles 16 and 17 of the Covenant (Addendum)* (28 February 2001) [12.02].

30 D Bilchitz, *Poverty and Fundamental Rights: The Justification and Enforcement of Socio-Economic Rights* (OUP 2007) 221; Tobin (n 7) 244.

31 Tobin (n 7) 243–45.

32 S Skogly, ‘The Requirement of Using the “Maximum of Available Resources” for Human Rights Realisation: A Question of Quality as Well as Quantity?’ (2012) 12 Human Rights L Rev 393, 400, 413–19.

33 ibid 404–9.

34 General Comment No 3 (n 12) [10].

35 S Fredman, *Human Rights Transformed: Positive Rights and Positive Duties* (OUP 2008) 85; GS McGraw, ‘Defining and Defending the Right to Water and Its Minimum Core: Legal Construction and the Role of National Jurisprudence’ (2011) 8(2) Loyola University Chicago Int L Rev 127, 156; S Fredman, *Comparative Human Rights* (OUP 2018) 71; DSK Maxwell, ‘Broadening the Human Rights Discourse, Realising Socio-economic Rights, and Balancing Rights to Property: Moving Beyond the Rhetoric of Socio-economic Rights and Scottish Land Reform’ [2019] Public L 121, 133.

36 General Comment No 14 (n 17) [47].

37 ibid.

38 ibid [52].

39 ‘The Maastricht Guidelines on Violations of Economic, Social and Cultural Rights’ (1998) 20 Human Rights Quart 691.

40 ibid [9].

41 ibid [15].

42 K Perehudoff and L Forman, ‘What Constitutes “Reasonable” State Action on Core Obligations? Considering a Right to Health Framework to Provide Essential Medicines’ (2019) 11 J Human Rights Practice 1, 10.

43 ibid.

44 DM Davis, ‘Socioeconomic Rights: Do They Deliver the Goods?’ (2008) 6 Int J Constit L 687, 688; S Germain, ‘Taking “Health” as a Socio-economic Right Seriously: Is the South African Constitutional Dialogue a Remedy for the American Healthcare System?’ (2013) 21 African J Int Comp L 145, 148.

45 Davis (n 44) 696; *Mazibuko* (n 20) [56]–[68].

46 MJ Hall and DC Weiss, ‘Human Rights and Remedial Equilibration: Equilibrating Socio-Economic Rights’ (2011) 36 Brooklyn J Int L 453, 466; Fredman (n 35) 244–45, 248–49.

47 See, eg A Pillay, ‘Economic and Social Rights Adjudication: Developing Principles of Judicial Restraint in South Africa and the United Kingdom’ [2013] Public L 599.

48 ibid 600.

49 ibid 602, 605; Germain (n 44) 150; see also M Amos, ‘The Second Division in Human Rights Adjudication: Social Rights Claims under the Human Rights Act 1998’ (2015) 15 Human Rights L Rev 549, 563.

50 Davis (n 44) 703; Pillay (n 47) 609–10; Amos (n 49) 563.

51 Pillay (n 47) 607–8.

52 Davis (n 44) 703.

53 Pillay (n 47) 604.

54 Forman (n 22) 74.

55 *Soobramoney v Minister of Health (Kwazulu-Natal)* 1998 (1) SA 765 (CC).

56 Pillay (n 47) 609.

57 Davis (n 44) 695; Germain (n 44) 157; Pillay (n 47) 604.

58 *Khosa v Minister of Social Development* 2004 (6) SA 505 (CC).

59 ibid [76].

60 *ibid*. [77].

61 ibid [62].

62 See, eg *R (McDonald) v Kensington and Chelsea Royal London Borough Council* [2011] 4 ALL ER 881 [16]; *Fok Chun Wa v The Hospital Authority* (2012) 15 HKCFAR 409 [10], [71]–[72]; *McDonald v United Kingdom* (2015) 60 EHRR 1 [54]–[55]; *R (Rotherham Metropolitan Borough Council) v Secretary of State for Business, Innovation and Skills* [2015] 3 ALL ER 1 [62]–[63].

63 S Fredman, ‘Human Rights Transformed: Positive Duties and Positive Rights’ [2006] Public L 498, 515 (emphasis added).

64 Fredman (n 35) 100, 103; Fredman (n 13) 307, 317–18.

65 See, for instance, the English law position in *Dover District Council v CPRE Kent* [2018] 2 ALL ER 121.

66 *R v Lyons* [2003] 1 AC 976 [27]–[28]; *Ubamaka Edward Wilson v Secretary for Security* (2012) 15 HKCFAR 743 [43]–[44]; E Lui, ‘The Right to Health in Hong Kong: Incorporation, Implementation, and Balancing’ in C Ó Néill, C Foster, J Herring and J Tingle (eds), *Routledge Handbook of Global Health Rights* (Routledge 2021) (forthcoming).

67 It is noteworthy that the conventional arguments against the justiciability of socio-economic rights have not precluded modern developments of socio-economic rights in the UK context: see K Boyle, ‘The First Minister’s Advisory Group on Human Rights Leadership: A New Path Forward on Incorporation of Economic, Social Cultural and Environmental Rights’ [2019] European Human Rights L Rev 361. A prominent example of such modern developments is the United Nations Convention on the Rights of the Child (Incorporation) (Scotland) Bill (SP Bill 80), which seeks to incorporate *both* civil and political and socio-economic rights (including the child’s right to health under art 24) under the United Nations Convention on the Rights of the Child into Scottish law: see United Nations Convention on the Rights of the Child (Incorporation) (Scotland) Bill, Explanatory Notes [5]. The Welsh developments are also notable: see Rights of Children and Young Persons (Wales) Measure 2011. Nevertheless, these developments are non-conclusive to the discussion here, since the objection applies beyond the UK context and is normative in nature (ie the critic may maintain that although these developments existed, they ought not to have occurred).

68 The argument made in this section is one for the *incorporation* of the right to life-saving emergency treatment in a dualist state. This is a distinct point from whether such a right should be directly enforceable at the international level, although the argument here would clearly have relevance for this distinct question. There are good reasons to exercise caution against conflating the justiciability of the right to life-saving emergency treatment at the domestic and international levels. For instance, there are important institutional differences—in the separation of powers – between domestic courts and international supervisory committees: see Fredman (n 35) 245.

69 N Jheelan, ‘The Enforceability of Socio-economic Rights’ [2007] European Human Rights L Rev 146, 147; Hall and Weiss (n 46) 466; Fredman (n 13) 301; Maxwell (n 35) 128.

70 ICESCR (n 1), art 15(1)(a).

71 Maxwell (n 35) 128.

72 J Wills, ‘The World Turned Upside Down? Neo-liberalism, Socioeconomic Rights, and Hegemony’ (2014) 27(1) Leiden J Int L 11, 20. See Optional Protocol to the International Covenant on Economic, Social and Cultural Rights (UN Doc A/63/435).

73 European Social Charter (Turin, 18 October 1961, entered into force 26 February 1965, 529 UNTS 89).

74 *Lopes* (n 17) [OI-24]. See, eg *European Roma and Travellers Forum (ERTF) v Czech Republic* (Complaint No 104/2014); *Conference of European Churches (CEC) v Netherlands* (Complaint No 90/2013).

75 European Committee of Social Rights, Statement of interpretation on the right to protection of health in times of pandemic (21 April 2020).

76 Jheelan (n 69) 147–48.

77 Nissen (n 3) 697.

78 *Lopes* (n 17) [190].

79 ibid [191].

80 ibid [192].

81 J Kapelańska-Pręgowska, ‘Medical Negligence, Systemic Deficiency, or Denial of Emergency Healthcare? Reflections on the European Court of Human Rights Grand Chamber Judgment in *Lopes de Sousa Fernandes v. Portugal* of 19 December 2017 and Previous Case-law’ (2019) 26 European J Health L 26, 43.

82 European Convention on Human Rights (Rome, 4 November 1950, entered into force 3 September 1953, 213 UNTS 221) art 4; International Covenant on Civil and Political Rights (New York, 16 December 1966, entered into force 23 March 1976, 999 UNTS 171) art 8.

83 *Rantsev v Cyprus* (2010) 51 EHRR 1 [284], [293]; *ZN v Secretary for Justice* (2020) 23 HKCFAR 15 [88], [92].

84 DWL Wang, ‘Social Rights Adjudication and the Nirvana Fallacy’ [2018] Public L 482, 483.

85 Fredman (n 63) 502; Jheelan (n 69) 154, 156.

86 Wang (n 84) 484.

87 ibid 484–85.

88 A Kavanagh, ‘Defending Deference in Public Law and Constitutional Theory’ (2010) 126 Law Quar Rev 222, 244–45.

89 Fredman (n 13) 298, 305, 317.

90 ibid 317.

91 ibid 305.

92 Nissen (n 3) 697.

93 *Lopes* (n 17) [175].

94 ibid [190].

95 This analysis also has further implications. There have been calls to the effect that within international human rights law, budget decisions should not merely be a matter of political choice. There have accordingly been suggestions for states to adopt a human rights framework—within which the discussion of socio-economic rights is integrated with budget analysis: see further C Harvey and E Rooney, ‘Integrating Human Rights? Socio-economic Rights and Budget Analysis’ [2010] European Human Rights L Rev 266; R O’Connell and others, *Applying an International Human Rights Framework to State Budget Allocations: Rights and Resources* (Routledge 2014). *Lopes* and Fredman’s analysis—as well as the minimum core and reasonableness approaches—are all possible components of such a framework.

96 General Comment No 3 (n 12) [10].

97 Ben King, ‘Coronavirus: How much will it cost the UK?’ (*BBC*, 22 October 2020) <https://www.bbc.com/news/business-52663523>; Press Association, ‘Furlough scheme extension: How Will it Work and What Will it Cost?’ (*Evening Express*, 5 November 2020) <https://www.eveningexpress.co.uk/news/uk/furlough-scheme-extension-how-will-it-work-and-what-will-it-cost/>

98 Skogly (n 32) 407.

99 BBC, ‘Coronavirus: Tens of Thousands of Retired Medics Asked to Return to NHS’ (*BBC*, 20 March 2020) <https://www.bbc.com/news/uk-51969104>

100 Ministry of Defence, ‘COVID Support Force: the MOD’s contribution to the coronavirus response’ (23 March 2020) <https://www.gov.uk/guidance/covid-support-force-the-mods-contribution-to-the-coronavirus-response#supporting-the-nhs>

101 General Comment No 14 (n 17) [47].

102 Denis Campbell, ‘NHS Short of Over £1bn for Covid Second Wave and Onset of Winter’ (*Guardian*, 26 October 2020) <https://www.theguardian.com/society/2020/oct/26/nhs-short-of-over-1bn-for-second-covid-wave-and-onset-of-winter>

103 Pillay (n 47) 609.

104 Nissen (n 3) 697.

105 J Herring, *Medical Law and Ethics* (6th edn, OUP 2016) 71. See also the discussion in E Wicks, *Human Rights and Healthcare* (Hart Publishing 2007) ch 2.

106 Nissen (n 3) 697.

107 Fredman (n 13) 303.

